# Linking altered central pain processing and genetic polymorphism to drug efficacy in chronic low back pain

**DOI:** 10.1186/s40360-015-0023-z

**Published:** 2015-09-16

**Authors:** Andreas Siegenthaler, Jürg Schliessbach, Pascal H. Vuilleumier, Peter Juni, Hanns U. Zeilhofer, Lars Arendt-Nielsen, Michele Curatolo

**Affiliations:** Chronic Pain Management, Lindenhof Hospital, Lindenhof Group Bern, Bern, Switzerland; University Department of Anesthesiology and Pain Therapy, Inselspital Bern, Bern, Switzerland; Institute of General Practice BIHAM, Faculty of Medicine, University of Bern, Bern, Switzerland; Institute of Pharmacology and Toxicology, University of Zurich, Zurich, Switzerland; Centre of Sensory Motor Interaction, University of Aalborg, Aalborg, Denmark; Department of Anesthesiology and Pain Medicine, University of Washington, Seattle, WA USA

## Abstract

**Background:**

Inability to predict the therapeutic effect of a drug in individual pain patients prolongs the process of drug and dose finding until satisfactory pharmacotherapy can be achieved. Many chronic pain conditions are associated with hypersensitivity of the nervous system or impaired endogenous pain modulation. Pharmacotherapy often aims at influencing these disturbed nociceptive processes. Its effect might therefore depend on the extent to which they are altered. Quantitative sensory testing (QST) can evaluate various aspects of pain processing and might therefore be able to predict the analgesic efficacy of a given drug. In the present study three drugs commonly used in the pharmacological management of chronic low back pain are investigated. The primary objective is to examine the ability of QST to predict pain reduction. As a secondary objective, the analgesic effects of these drugs and their effect on QST are evaluated.

**Methods/Design:**

In this randomized, double blinded, placebo controlled cross-over study, patients with chronic low back pain are randomly assigned to imipramine, oxycodone or clobazam versus active placebo. QST is assessed at baseline, 1 and 2 h after drug administration. Pain intensity, side effects and patients’ global impression of change are assessed in intervals of 30 min up to two hours after drug intake. Baseline QST is used as explanatory variable to predict drug effect. The change in QST over time is analyzed to describe the pharmacodynamic effects of each drug on experimental pain modalities. Genetic polymorphisms are analyzed as co-variables.

**Discussion:**

Pharmacotherapy is a mainstay in chronic pain treatment. Antidepressants, anticonvulsants and opioids are frequently prescribed in a “trial and error” fashion, without knowledge however, which drug suits best which patient. The present study addresses the important need to translate recent advances in pain research to clinical practice. Assessing the predictive value of central hypersensitivity and endogenous pain modulation could allow for the implementation of a mechanism-based treatment strategy in individual patients.

**Trial registration:**

Clinicaltrials.gov, NCT01179828

## Background

Drug therapy is an essential part of chronic pain treatment. However, only a minor part of pain patients sufficiently benefits from the available treatments or is able to tolerate the drugs. One important limitation of drug therapy is lack of instruments to predict their effect. For this reason, patients are often prescribed medications on a trial-and-error basis. Several attempts are typically required until an appropriate treatment can be installed. In clinical practice “classes” of drugs (e.g. antidepressants) are given to “classes” of patients (e.g. neuropathic pain patients). However, within those classes of patients, very different pain mechanisms are likely to underlie the pain condition in different individuals. Drugs that affect part of these mechanisms will therefore probably not work in all patients. Another reason for variability in drug responses is genetic variation, leading to a spectrum of different responses to analgesics, including lack of efficacy, exaggerated response or even intolerable adverse effects.

Quantitative sensory testing (QST) comprises methods that assess alterations and reorganization of the nociceptive system. Presence of an abnormal measurement in a chronic pain patient could provide us with the information that the underlying pain pathways might somehow be altered [1]. An essential question is whether this information can be linked to drug efficacy in a mechanism-based treatment approach. QST has already been shown to predict the efficacy of duloxetine in painful diabetic neuropathy [2] and of pregabalin in chronic pancreatitis [3]. For one of the most common chronic pain disorders, namely chronic low back pain, such investigations have not yet been reported.

Genetic variations such as polymorphisms of drug metabolizing enzymes affect drug response as well. A further important question is therefore whether assessing genetic polymorphisms before initiating pharmacological treatment can explain different drug effects and hence help selecting the appropriate therapeutic strategy for individual patients.

### Objective

Three drugs are administered in three cohorts of patients suffering from chronic low back pain: the tricyclic antidepressant imipramine, the opioid agonist oxycodone and the GABA_A_-agonist clobazam.

The primary aim of this study is to investigate whether quantitative sensory tests measured before drug administration can predict the analgesic efficacy of a single oral dose of each of the three drugs.

The secondary aim is to further investigate the effect of each drug on the intensity of chronic low back pain and to analyze how quantitative sensory tests are influenced by each drug over time. Genetic factors affecting drug metabolism and pain sensitivity will be analyzed as additional explanatory variables for drug efficacy.

### Status

**Fig. 1:**
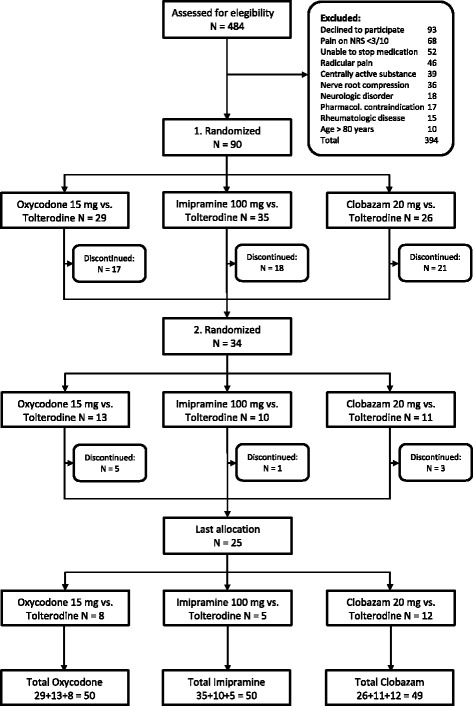
Presents the flow of participants through the study. From the initially included 90 patients, 34 decided to participate in a second session, the remaining 56 declined further participation. Twenty-five of the 34 re-randomized patients wished to participate in the third session. This resulted in an overall allocation of 50 patients to both the oxycodone and imipramine arm and 49 patients to the clobazam arm. NRS = numeric rating scale (0 = no pain, 10 = worst pain imaginable)

The study is currently running. Recruitment has been completed, but no data have yet been published and no paper has been submitted. Since several publications are expected to result from this project, we present this protocol as a future reference in order to improve the readability of the resulting studies and to describe in detail the methods, design and allocation processes that are being used. The flow of patients through the study is depicted in detail in the flowchart in Fig. 1.

## Methods

### Design

This randomized placebo-controlled trial in consecutive patients with chronic low back pain is carried out at the University Department of Anesthesiology and Pain Therapy of the Inselspital Bern, Switzerland. The study is approved by the local ethics committee (KEK 213–09), registered with clinicaltrials.gov (NCT01179828) and strictly follows good clinical practice guidelines and the Helsinki declaration.

Primarily, the data of each drug are analyzed separately, resulting in three sub-studies. Patients will be randomly assigned to one of them. These three sub-studies have the same aims, use the same experimental procedures and statistical analyses. The only difference among the three sub-studies is the drug investigated. The inclusion of the three sub-studies in the same protocol and the random allocation of patients to the sub-studies may allow secondary exploratory analyses comparing the effects of the three drugs. The active placebo tolterodine is used in order to control for a possible placebo effect of the investigated drugs [4–6].

### Patients

We test consecutive patients with chronic low back pain referred to the Pain Division or recruited by advertising. Chronic low back pain is defined as the presence of low back pain on most days for the duration of three months or longer [7, 8]. Any regular pain medication is stopped one week prior to the first experiment. During the study period, only acetaminophen and ibuprofen are allowed as rescue medication, but they must as well be stopped 24 h before a testing session. Patients unable to stop their analgesic regimen because of pain exacerbation are not included in the study.

### Exclusion criteria are:

- Age less than 18 or more than 80 years
- Pain intensity at rest < 3/10 on the numerical rating scale (NRS) at the time of testing, whereby 0 = no pain and 10 = worst pain imaginable
- Suspected radicular pain, as defined by leg pain associated with an MRI finding of a herniated disc, or foraminal stenosis with contact to a nerve root
- Signs or suspicion of neurological dysfunction at the tested sites
- Pregnancy or breast feeding as assessed by pregnancy test
- Ongoing treatment with an antidepressant, opioid or benzodiazepine or intake of other centrally active substances (including drug or alcohol abuse)
- Known allergy or pharmacological contraindications to any of the tested substances
- Multi-site or widespread pain as well as systemic inflammatory or rheumatological disease
- Major depression (Beck depression inventory short form score >9)

Since all the patients are randomly assigned to one of the 3 study groups, pharmacological contraindications for all 3 substances have to be considered. Elevated eye pressure, obstructive uropathy, severe heart disease and documented QTc prolongation, documented or suspected ischemic heart disease are special contraindications for the use of imipramine. Severe pulmonary disease with or without heart failure, hypoxemia, neuromuscular disease (e.g. myasthenia gravis) and neurologic disease are additional contraindications for opioids and to a lesser extent for clobazam. History of lack of effectiveness or known occurrence of intolerable side effects after intake of one of the study drugs are exclusion criteria as well.

### Drugs investigated

#### Imipramine

Antidepressant drugs have been used for many years to treat chronic pain patients, especially suffering from neuropathic pain, independent of a concomitant depressive disorder. For classical tricyclic antidepressants, the number-needed-to-treat (NNT) for chronic neuropathic pain is approximately 3 for moderate pain relief [9]. Antidepressants are also effective in musculoskeletal pain conditions [10–12], possibly because of their effect on the endogenous modulating mechanisms involving the monoaminergic system [12]. A single oral dose of imipramine 100 mg was effective in studies using experimental pain tests in healthy volunteers [13, 14]. Considering that our patient population would be older, we choose a dose of 75 mg.

#### Oxycodone (immediate release)

Oxycodone is an opioid agonist largely used for different chronic neuropathic, musculoskeletal and neoplastic pain conditions. It may be effective in chronic musculoskeletal pain [15]. A single oral dose of oxycodone 15 mg is administered in this study. This has been shown to effectively modulate multimodal experimental pain in healthy volunteers without causing major adverse effects [16].

#### Clobazam

Inflammatory and neuropathic conditions cause reduced nociceptive control within the spinal cord via inhibition of the GABA_A_-receptor [17], leading to pain facilitation. Benzodiazepines produce antinociception in animal pain models by acting on the GABA_A_-receptor [18] and are, therefore, of potential interest in human pain management as well. The benzodiazepine that is most commonly prescribed in pain treatment is clonazepam. However, sedation strongly limits its clinical usefulness. Clobazam is another GABAergic compound that causes less sedation than clonazepam [19], making it a potentially interesting drug in the clinical management of pain. Studies on its efficacy in chronic pain are lacking. A dose of 20 mg is chosen, corresponding to an equivalent anticonvulsant dose of 0.5 mg clonazepam.

#### Active placebo: tolterodine

All of the evaluated drugs are likely to be associated with minor central side effects, such as dizziness or sedation. While these side effects are only transient and irrelevant for the patient safety overall, they can potentially impair blinding of patients and investigators. Therefore, an active placebo has to be chosen that produces similar central side effects, but has no analgesic effectiveness. Tolterodine is an anticholinergic compound prescribed in hyperactive bladder, which, as other anticholinergic compounds, causes some sedation and dry mouth, but is devoid of analgesic effects. The starting dose usually recommended in the Swiss Drug Compendium is 2 mg twice a day, which can be decreased to 1 mg twice a day. In order to minimize the likelihood of excessive side effects, a dose of 1 mg is given in this study.

#### Concealment of allocation and blinding

Randomization is performed by the hospital pharmacy in blocks of 6 based on computer generated random numbers. Tablets are concealed in red colored gelatin capsules (LGA, France) and packed into semi-opaque flasks labelled with the patient and session number, lot number and expiry date. All processes strictly follow good manufacturing practice (GMP) guidelines. Both investigators and patients are blinded as to the sequence of application of the study drug and placebo. Patients are allowed to participate in more than one drug arm if they wish. These cases are announced prospectively to the pharmacy, which ensures that the patient is not accidentally allocated to the same sub-study in which he already participated earlier. This procedure does not negatively influence randomization or blinding. Patients participating in more than one sub-study repeat the active placebo session for each additional drug arm.

### Mechanisms assessed

All three drugs may affect endogenous pain modulation (i.e. endogenous mechanisms that attenuate or enhance nociceptive processes) and hypersensitivity (i.e. increased sensitivity to pain that is typically associated with chronic pain states [20]). These disturbances will therefore be the primary predictive variables for drug efficacy. Control of nociceptive processes by the endogenous inhibitory system is evaluated by conditioned pain modulation (CPM) and generalized hypersensitivity is assessed by pressure and electrical pain thresholds.

Genetic polymorphisms may either affect pain sensitivity in general or the metabolism of the investigated drugs. We therefore genotype the following cytochromes: CYP2C19 (involved in imipramine and clobazam metabolism), CYP2D6 (involved in imipramine and oxycodone metabolism) and CYP3A4 (oxycodone and clobazam metabolism) [21, 22]. The μ-opioid receptor variant A118G (oxycodone binding site) is genotyped in the oxycodone arm [23]. Polymorphisms of pain genes known to alter responses to experimental pain are determined in all patients: COMT (catechyl-o-methyltransferase [24]); GCH-1 (GTP-Cyclohydroxylase [25]) and the potassium channel subunit KCNS1 [26]. Genotyping is performed using real-time PCR and identification of specific variants by means of melting curve analysis. A list of the specific polymorphisms that are analyzed can be found in table 1.

**Table 1:** List of polymorphisms examined

KCNS1		rs734784
GCH-1		rs8007267
		rs3783641
		rs10483639
OPRM	A118G	rs1799971
COMT		rs6269
		rs4633
		rs4818
		rs4680
CYP3A	3A4*1b	rs2740574
	3A4	rs4646437
	3A5*3	rs776746
CYP2D6	CYP2D6*6	rs5030655
	CYP2D6*7	rs5030867
	CYP2D6*8	rs5030865
	CYP2D6*10	rs1065852
	Cyp2D6*41	rs28371725
	CYP2D6*3A	rs35742686
	CYP2D6*4	rs3892097
	CYP2D6*5	Complete deletion of gene
	CYP2D6*2	Duplication; more than one *2 variant
CYP2C19	CYP2C19*1	n/a
	CYP2C19*2	rs4244285
	CYP2C19*3	Rs4986893

### Quantitative sensory tests

During the experimental session, patients are positioned in a comfortable supine position, with the upper body elevated by 30°, in a closed and quiet room. Before starting the experiment, training sessions of the pain tests are performed until the subjects are familiar with the testing procedures. All QST measurements are performed on the more painful body side. In case of bilateral or midline pain, the side is randomly selected by drawing lots. All QST measurements are performed at baseline and 1 and 2 h after drug administration. Triplicate measurements are made for all tests except for conditioned pain modulation and train-of-twenty stimulation (see below).

#### Pressure pain thresholds

Pressure pain detection and tolerance thresholds (PPDT and PPTT, respectively) are measured with an electronic pressure algometer (Somedic AB, Horby, Sweden) [27] applied at the center of the pulp of the 2^nd^ toe. The probe has a surface area of 1 cm^2^. The pressure is increased from 0 at a rate of 30kPa/s to a maximum pressure of 1000kPa. Pain detection threshold is defined as the point at which the pressure sensation turns to pain. Pain tolerance threshold is defined as the point at which the subject feels pain as intolerable. The subjects are instructed to press a button when these points are reached. If the subjects do not press the button at a pressure of 1000 kPa, this value is considered as threshold.

#### Single and repeated electrical stimulation

Electrical single and repeated pain thresholds (ESPT and ERPT, respectively) are performed using a computer-controlled constant current stimulator (Digitimer Ltd, England). Bursts of five 1 ms square wave impulses within 25 ms (perceived as one single stimulus) are delivered via two Ag-AgCl electrodes placed distal to the lateral malleolus in the innervation area of the sural nerve. The current intensity is increased from 1 mA in steps of 1 mA until the sensation becomes painful (ESPT). For ERPT, the stimuli are repeated five times at a frequency of 2 Hz. Current intensity of all 5 stimuli are increased in steps of 1 mA until the last 2–3 stimuli are perceived as painful, corresponding to the temporal summation threshold.

#### Heat and cold pain thresholds

Temperature pain thresholds are assessed using a peltier thermode (TSA II, Medoc, Israel) with a probe surface of 3x3 cm. Starting at 30.0 °C, the temperature is changed at a rate of 1 °C/s. Subjects will stop the measurements by pressing a button when the warm sensation turns to pain (heat pain detection threshold, HPDT) or when it becomes intolerable (heat pain tolerance threshold, HPTT), or when the cold sensation starts to be painful (cold pain detection, CPDT). In any case, the measurements will be stopped at 50.5 °C for HPTT or 0 °C for CPDT, respectively. Measurements are made at the lateral aspect of the lower leg (dermatome L5) and the radial surface of the proximal forearm (dermatome C6).

#### Conditioned pain modulation (CPM)

This method explores the endogenous modulation of nociceptive input. Under normal conditions, pain after application of a test stimulus is attenuated by the application of an additional “conditioning” stimulus to a remote body region [28, 29]. The cold pressor test will be used as the conditioning stimulus. Subjects will immerse their contralateral hand into ice-saturated water (1.5 ± 1 °C) until they rate the cold pain as 7/10 on the NRS. During the baseline measurements, a single assessment of PPDT is performed as test stimulus. An increase in PPDT compared to the measurement before hand immersion is an indication of CPM.

Electrical repeated stimuli are as well used as a test stimulus for CPM. A train-of-five electrical stimulus at an intensity of 1.2 times the ERPT is rated by the subject on a 0–10 NRS before and during the cold pressor test. A decrease in pain rating during the cold pressor test is another indication of CPM. These assessments will be performed at baseline as well as 1 and 2 h after drug intake in order to study the influence of the drug on endogenous pain modulation.

#### Electrical train-of-twenty stimulation

The arithmetical mean of three ERPT assessments at baseline are used to deliver 20 identical stimuli over 10 s with a frequency of 2 Hz. This stimulus intensity remains constant over the two subsequent measurements 1 and 2 h after drug intake. Subjects rate the maximal and final pain intensity during this stimulation on a 0–10 NRS. A decrease in pain intensity in the subsequent measurements would be indicative of an analgesic effect and a difference between maximal and final pain intensity during the 20 stimulations is considered a feature of endogenous pain modulation.

#### Pain intensity, side effects and global impression of change

The intensity of low back pain is rated by the patient on a 0–10 NRS in both the sitting and supine position. The occurrence of nausea, vomiting, sedation and dizziness are also recorded on 0–10 NRS, whereby 0 = no side effect at all and 10 = extreme occurrence of side effect. Any other reported side effect will be recorded. The patients’ global impression of change scale (PGIC) consists of a 7-point Likert scale ranging from 1 = “very much improved” over 4 = “no change at all” to 7 = “very much worse”. Pain intensity, side effects and PGIC will be assessed in intervals of 30 min up to two hours after drug intake.

### Outcome parameters

For the primary objective, which is to predict analgesic efficacy of each drug, pain intensity on a 0–10 NRS in the supine position after 2 h is the primary outcome parameter. Pain in the sitting position and PGIC will be secondary outcome parameters. Baseline QST parameters, genetic variants and side effects will be used as explanatory (independent) variables.

For the secondary objective, which is to document the analgesic effect of each drug, the primary outcome parameter will again be the intensity of low back pain in the supine position on a 0–10 NRS. All QST parameters will be secondary outcome variables.

#### Descriptive variables

The following descriptive variables will be recorded: age, gender, body mass index, pain intensity at time of testing, average pain intensity in the last 24 h before testing (0–10 NRS), duration of pain in months, history of surgery due to painful condition (yes/no), pain-related life interference as assessed by the Multidimensional Pain Inventory (MPI) [30], Catastrophizing scale [31] and the Beck Depression Inventory (BDI) short form [32].

### Sample size considerations

Based on a correlation of pain scores across active and placebo phase within a patient of 0.65, a prevalence of treatment responders of 40 %, an average difference of pain reduction measured with the NRS-scale between the active drug and placebo of 2.5 in the treatment responders and 0 in “treatment non-responders”, 50 patients per sub-study will be required to detect an interaction between treatment effect and QST-status at a two-sided alpha-level of 5 % with a power of 90 %.

### Data analysis

Repetitive assessments of pain intensity (NRS, primary endpoint) and Patients Global Impression of Change Scale (secondary endpoint), recorded 30, 60, 90 and 120 min after intake of active treatment or placebo, will be analyzed using a multilevel model adjusted for baseline values, with random effects at the level of patients and test phases (verum vs. placebo). Analyses will be stratified according to QST-status and pharmacogenomic characteristics. A formal test of interaction between treatment, QST and pharmacogenomic status will be performed. The primary and secondary endpoints, as well as the primary and secondary predictive variables are described above in page 17/18 and 11, respectively. Data validation, query management and analysis will be performed by CTU Bern. The data analyst will be blinded as to the allocated interventions for primary analyses. *P*-values are 2-sided. Analyses will be performed in Stata 10 (Stata Corporation, College Station, Tex).

## Discussion

Identifying the best drug in a given patient is a time consuming and frequently frustrating task for all the persons involved. The growing knowledge about pain perception and transmission has led to a new understanding of how chronic pain might be maintained and offers several new targets for therapeutic management. Such mechanism-based treatment approaches are given high priority in translational pain research. The combination of phenotyping disturbances in pain processing and genotyping predisposition to pain and polymorphism in drug metabolism may offer the perspective of a more specific treatment of chronic pain. This may ultimately lead to a better selection of the therapeutic strategy in individual patients, thereby reducing the likelihood of ineffective treatment and adverse effects.
